# Benefit of an action camera in endoscopy education for medical students under COVID-19

**DOI:** 10.1186/s12909-023-04702-6

**Published:** 2023-09-22

**Authors:** Akira Uchiyama, Shunhei Yamashina, Toshifumi Sato, Satoshi Sakuma, Yuichi Tomiki, Hiroyuki Isayama, Akihito Nagahara, Kenichi Ikejima

**Affiliations:** 1 Department of Gastroenterology, Juntendo University School of Medicine, 2-1-1 Hongo, Bunkyo-Ku, Tokyo, 113-8421 Japan; 2 Medical Education, Juntendo University School of Medicine, Tokyo, Japan

**Keywords:** GoPro, Live- action camera, COVID-19, Esophagogastroduodenoscopy, Medical education

## Abstract

**Background:**

Endoscopy is an important form of clinical gastroenterology education because it gives students the opportunity to learn about diagnosis procedures and even treatment. During the COVID-19 pandemic, medical students were observed from outside the endoscopy room due to the risk of airborne infection. In this study, we investigated the efficacy of combining endoscopy education with doctor’s-eye-view videos of the procedure obtained using live-action cameras (GoPro®).

**Methods:**

From February to May 2021, endoscopists wore GoPro Hero8 cameras on their heads to display a doctor’s-eye view video outside the room. The efficacy of the GoPro videos in combination with endoscopic monitoring was evaluated by 15 participating medical students. The participants rated the efficacy on a 5-point scale and commented on the positive and negative points.

**Results:**

A total of 78.6% of participants evaluated the GoPro as good; 57.2% answered that it increased their understanding, with 71.4% stating that it increased their understanding of procedures in particular. A total of 85.7% of the students answered that their interest in endoscopy had increased, and 85.7% evaluated the benefit of the GoPro videos as good. In addition, 64.3% answered that the method was effective in preventing COVID-19 infection. Education using GoPro videos enabled students to feel as if they were conducting the endoscopy themselves and enabled them to concentrate on learning.

**Conclusions:**

Practical endoscopic education using a GoPro is an effective educational tool that not only increases understanding of endoscopic practice but also stimulates students’ interest and awareness of their future as doctors.

## Background

In clinical medical education, hospital training is a valuable opportunity for students to learn not only the medical practices of clinicians but also how to interact with patients and cooperate with healthcare professionals [1, 2]. Medical students are trained in clinical skills, including medical examination techniques, and in specific clinical skills through patient care under the guidance of a supervisor [3]. However, medical student can only perform a limited number of medical interventions themselves, as many medical interventions can only be performed after practitioners have passed the national medical examination and have a specialty. Although some clinical procedures can be learned from simulated patients and practice equipment, the variety is limited, and it is difficult to study many medical procedures performed in the hospital setting. While training in the department of gastroenterology, medical students study abdominal examinations and patient management in hospital units. They also study abdominal ultrasonography and esophagogastroduodenoscopy (EGD). Non-invasive examinations, such as ultrasonography, can be performed by medical students, and students can practice on one another as well as on simulation devices. However, EGD is an invasive procedure that cannot be performed by students. Because medical students can only observe the procedure, they cannot get a real impression of it.

The novel coronavirus disease 2019 (COVID-19) became a rapidly spreading global pandemic in 2020 that attracted worldwide attention. On 30 January 2020 the International Health Regulation 2005 Emergency Committee declared the COVID-19 outbreak a public health emergency of international concern (World Health Organization [WHO], 2020a). The Japanese government declared a state of emergency in April of 2020, which was lifted in late May [4]. During this time, many clinical clerkships were cancelled because young people could be asymptomatically infected with the virus and could infect patients with whom they came into contact during their clinical clerkships [5–7]. All lectures, except for clinical practice, were provided online. Students were kept out of hospitals, and non-essential outings were restricted to prevent COVID-19 infection [8]. Endoscopy is an essential part of gastroenterological clinical training. In this training, students learn not only endoscopic techniques and findings but also how doctors care for their patients. However, EGD observation during clinical training was also cancelled to prevent infection [9, 10], so students had to learn through virtual devices and videos [11]. The COVID-19 outbreak led to the development of various virtual devices and online educational systems [11], but this educational environment without face-to-face patient contact is thought to have taken away the opportunity for medical students to become aware of themselves as doctors. As measures to prevent COVID-19 infection became clearer, clinical practice in hospitals has now reopened. However, to prevent spread, social distancing is still required, and contact time between students and patients remains restricted [12]. Before COVID-19, medical students entered the endoscope room and observed alongside the endoscopist; after COVID-19, the practice changed to viewing the EGD monitor from outside the examination room. This prevents infection, but using the monitor makes it difficult to learn about patient care during the examination. Moreover, students cannot observe the cooperation between the endoscopist and the EGD assistants. A head-mounted ultra-high-definition video (GoPro®) has been reported to be useful as an educational device for observing surgical techniques and narrow-field-of-view medical procedures from the doctor’s point of view [13, 14].

Therefore, the aim of this study was to evaluate the efficacy of using both EGD monitor screens and doctors’-point-of-view images from GoPro cameras in EGD training.

## Methods

### Study Design and materials

The study was conducted with students on clinical training in the Gastroenterology unit of Juntendo University Hospital between February and May 2021. Participating students were all students who rotated during that period.

**Fig. 1:**
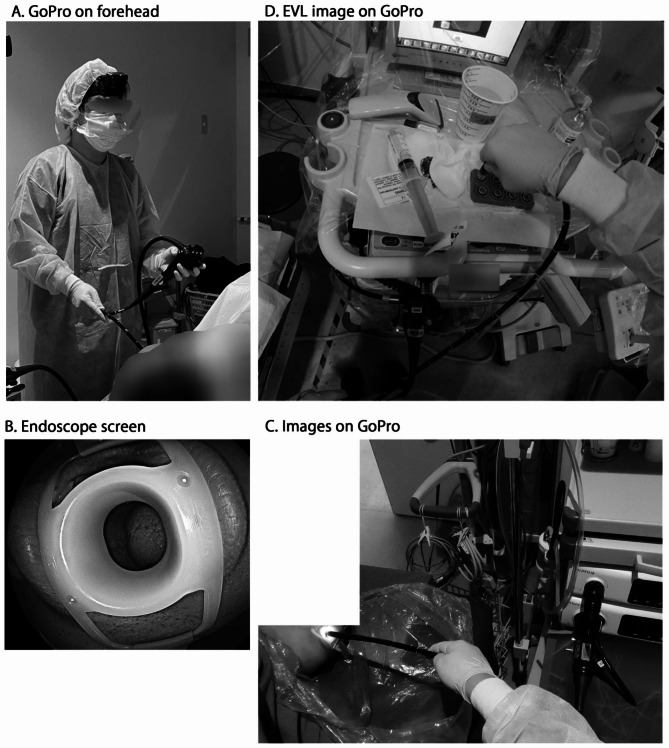
GoPro-images as the doctor’s point of view

The endoscopist used a GoPro® Hero8 and HEADSTRAP (San Mateo, CA, USA) to send a doctors’-point-of-view video to a 12-inch tablet device (Apple iPad Pro®, Apple Inc., Cupertino, USA), using a wireless connection (Fig. 1A). The endoscopists in this study were specialists and supervisors with at least 15 years of experience in endoscopic procedures.

First, medical students studied routine and therapeutic endoscopy from outside the endoscope room by observing an endoscopic video (Fig. 1B) narrated by the physician. The endoscopist’s voice is heard outside the endoscopy room via speakers and directly in this study. Especially during biopsies and treatment of varicose veins, the endoscopist explained why the biopsy was being performed and during endoscopic variceal ligation (EVL) procedures, the endoscopist showed images via a GoPro screen on the EVL device (Fig. 1D). Next, a GoPro live video was added for endoscopic education (Fig. 1 C and 1D), after which medical students were asked to fill out questionnaires on the experience.

### Evaluation of GoPro-combined education

To evaluate the efficacy of the GoPro in teaching about endoscopy practice, students were asked to complete questionnaires after the procedure The questionnaire covered six topics: (1) comprehension of EGD, (2) doctor’s point of view, (3) comprehension of EGD techniques, (4) interest in EGD, (5) protection against infection, and (6) practical learning with GoPro. These were evaluated on a 5-point scale (unacceptable, poor, fair, very good, and excellent). In addition, we collected student’s opinions on the good and bad points of GoPro dual-use education.

## Results

### Evaluation of endoscopy education with GoPro in medical education

**Fig. 2:**
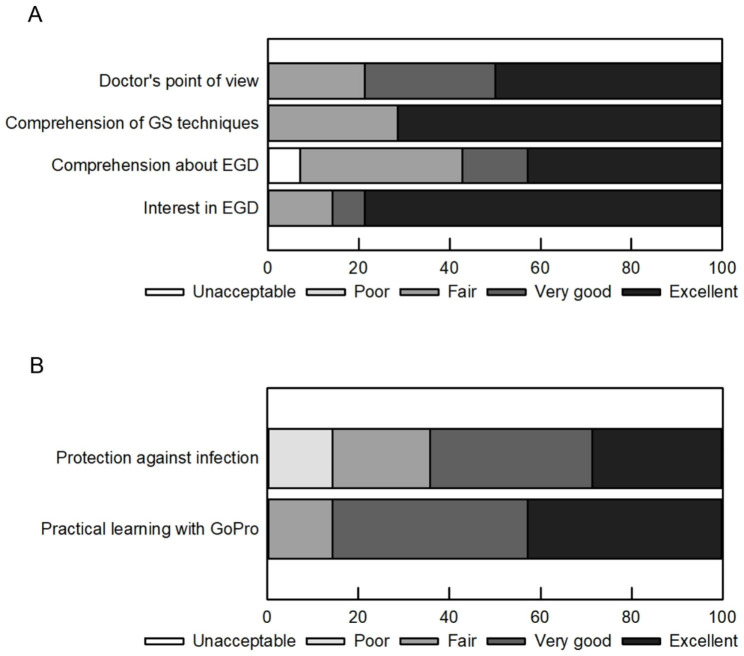
Evaluation of GoPro-combined education

This study evaluated the efficacy of GoPro videos of EGD practice for 15 medical students. When asked about the co-use of the GoPro video from the doctor’s point of view, 50% of the students answered ‘Excellent’, and 28.6% answered ‘Very Good.’ Regarding their comprehension of EGD techniques, 72.4% of the students answered ‘Excellent’, and none of the students evaluated it poorly. Regarding their comprehension of EGD, 42.9% of the students answered ‘Excellent’, and 14.3% answered ‘Very Good’. On the question regarding students’ interest in EGD, 78.6% of answered ‘Excellent,’ and 7.1% answered ‘Very Good’, while 14.3% answered ‘Fair’ (Fig. 2A). Regarding prevention of infection through the GoPro combination practice, 28.6% of the students answered, ‘Excellent’, 35.7% answered ‘Very Good’, 21.4% answered ‘Fair’, 14.3% answered ‘Poor’. In terms of overall evaluation of the hands-on training with the GoPro. 42.6% rated it as ‘Excellent’ and 42.6% stated ‘Very Good’ (Fig. 2B).

### Comments on GoPro combination education

In addition to the questionnaire, the students noted the good and bad points of the GoPro procedure. On the positive side, students answered that it was better to use the GoPro video in addition to the endoscopy monitor-only training, so that they could learn not only about endoscopic findings but also experience the overall examination and activity around the physician. Students also stated that the GoPro video made it feel as if they were performing the procedure themselves, and it made it possible for them to concentrate on the learning process. They were also able to learn how to work with medical assistants during EGD.

As a bad point, they pointed out the narrow field of view of the GoPro, and the endoscopist had to move the face over to widen the field of view. On the other hand, some said that if the GoPro is moved too much to widen the field of view, the image on the iPad moves so rapidly that it causes screen sickness. The narrow field of view of the GoPro makes it impossible to operate the endoscope, especially to observe the air delivery and inhalation buttons and the angle section. Depending on the Wi-Fi environment, GoPro images were sometimes difficult to view on the iPad. (Table 1)

**Table 1:** Comments from students on endoscopy observation with GoPro

**Good points**
It was possible to observe the examination from the beginning to the end.
It was possible to observe preparations other than during the examination through the GoPro video.
It was possible to observe the endoscopic technique and the realistic feeling of inserting the endoscope into the patient at the start of the endoscopy.
It was possible to concentrate on the examination with a realistic atmosphere.
It is great to see the patient’s facial expressions during the examination.
It was good to feel like being examined by myself.
Understanding of the cooperation with medical assistants.
In addition to the endoscopy views, it was good to be able to observe the preparation for examinations and procedures from the doctor’s point of view. Observation of the doctor’s view during biopsy forceps delivery, EVL preparation, and over-tube insertion.
It was possible to observe the details, such as the care for patients.
Wish the GoPro would be used to observe other examinations as well.
**Bad points**
GoPro video does not reflect the endoscopist’s face without moving it.
GoPro screen moves so quickly that it causes screen sickness
Sometimes the hands cannot be seen unless the endoscopist moves his or her face. The narrow field of view visible in the GoPro image The left handling of the endoscope is outside the GoPro’s field of view and therefore it cannot be observed.
Internet and Wi-Fi environment for GoPro connection

## Discussion

COVID-19 is highly contagious, and the pandemic required facilities to develop infection control strategies [15]. In education, classroom group lectures were cancelled and switched to online training and assignments [16]. As the infection control strategy against COVID-19 was gradually established [12], all medical students in our hospital resumed clinical clerkship after their temperatures and physical conditions were checked, following the infection control strategy. However, younger people, including medical students, could be asymptomatically infected with COVID-19, and in clinical clerkship, they had to be careful to avoid contact with immunocompromised patients, patients with respiratory diseases, and intensive care patients [7].

EGD can cause patients to cough, and there is a risk of airborne infection in the endoscopy room. Infection can be caused not only by droplets and contact but also by aerosolised COVID-19 [17]. Of the 623 asymptomatic patients scheduled for endoscopy, six tested positive for COVID-19 [18]. Patients remove their masks for the EGD, so patients, physicians, and medical assistants in the endoscopy room are increasingly at risk of infection. We have reported the effectiveness of endoscopic shields in preventing aerosol droplets during EGD examinations [19]. However, shielding alone does not completely prevent infection. In brief, EGD is a high-risk examination in terms of infection, and the room must be completely infection controlled [20].

Before COVID-19, endoscopy was a highly specialised technique and a popular lecture for students. After COVID-19, medical students were only able to observe the procedure through endoscopic monitors from outside the endoscopy room, making it difficult to provide adequate education. The purpose of this hands-on training with GoPro was to provide students with a better understanding of the endoscopic procedure while also controlling infection (Fig. 2A and B). In EGD education from outside the endoscopy room, only the endoscopic video and the doctor’s voice during the examination can be observed. During insertion, only the mouth is visible. However, with a GoPro video, students can observe the patient’s facial expression and the timing of the endoscope insertion (Fig. 1C). In addition to checking the endoscopic monitor, endoscopists check the electrocardiogram and oxygen monitors as needed before and during the examination, and they collaborate with the medical assistants in the room (Fig. 1D). GoPro makes it possible to observe this as well, and it is considered to be a great educational tool for medical students. Of the participating students, 78.6% evaluated the GoPro video with high scores. In addition, 71.4% of the medical students answered that it was possible to observe the operations at hand on the GoPro screen, and 71.4% of medical students evaluated it highly in terms of their education on endoscopic procedures (Table 1; Fig. 2A). In fact, it is difficult to observe the patient’s facial expression and the atmosphere during endoscopy from the physician’s standing position using only an endoscope monitor.

In this endoscopic education with GoPro, the students observed the scene where the biopsy forceps are being given by the caregiver during the biopsy and the physician’s view after the biopsy was performed. In particular, during the treatment of varicose veins (endoscopic variceal ligation: EVL), in addition to the usual endoscopic observation, the students were able to observe the physician preparing for EVL, attaching the EVL device, inserting the overtube into the patient, and removing the overtube after EVL. The use of realistic video images had the effect of making it seem as if the students were performing the examination (Table 1). Among the medical students, 56.9% answered that their comprehension of endoscopy increased, and 85.7% of students answered that they were interested in endoscopy (Fig. 2A). Furthermore, students could use both the endoscopic video and GoPro video while monitoring the endoscopist’s explanations, which was an effective result. Regarding the endoscopy time, it was no different from the usual endoscopy and procedures, and the use of the GoPro did not affect the examination time.

GoPro is an effective tool for endoscopy education. As for infection control, 64.3% of medical students evaluated it highly, but some students answered that it was inferior to being fully online because they had to enter the endoscopy centre, although they did not enter the endoscopy room (Fig. 2B). For this study, the GoPro video was sent to iPad screens wirelessly for observation. Some students commented on problems with the wireless connection and screen sickness. (Table 1) There were many opinions about the narrow field of view visible in the GoPro image and the Wi-Fi environment as education on the use of GoPro together. In particular, if the GoPro image from the forehead had a wide angle image, it would have been possible to see from the patient’s face to the operation at hand.

Some students did not appreciate the left-hand controls of the endoscope, such as the air delivery and suction buttons and angles, because they were outside of the GoPro image and could not be seen. It is necessary for endoscopists to understand the range of viewable areas on the GoPro, and to improve the GoPro in the future. By improving these problems, multiple students can observe during an examination at the same time.

There have been several reports on medical education using GoPro. COVID-19 infection prevention prevented many face-to-face education opportunities, and the usefulness of remote education using wearable cameras, including GoPro, has also been reported [21]. In particular, the use of GoPro in surgical procedures has been reported to improve understanding and technique [13, 14]. Endoscopic education such as hands-on seminars and other endoscopic education and mainly explain lesions on an endoscopic monitor. However, multiple assistants are present during endoscopic procedures, and endoscopists do not only observe the endoscopic monitor. This is the first report on the usefulness of the GoPro combination in endoscopic education. Education from the physician’s perspective is considered to be an important process, especially for medical students.

In addition, although there are privacy protection issues, students who must stay home from clinical practice because of illness can also share their observation practice online.

At our hospital, the endoscopy centre performs a variety of endoscopic examinations, including EGD, colonoscopy, device assisted enterosopy, endoscopic retrograde cholangiopancreatography related procedures, diagnostic and therapeutic endoscopic ultrasonography, and endoscopic submucosal dissection for malignant tumours. In this study, we used GoPro combined with education on EGD, but students requested that various other endoscopic examinations also be performed with GoPro (Table 1). Furthermore, by recording endoscopic videos and GoPro videos of emergency cases, such as gastrointestinal bleeding, which are not usually encountered during educational programs, students will be able to learn endoscopic operations from the doctor’s point of view and observe cooperation with medical assistants in the endoscopy room. Endoscopic education with GoPro was performed while sufficient education was not possible due to the outbreak of COVID-19. Considering the responses from students, even after the end of COVID-19, the GoPro combination is an excellent educational style and will continue to be introduced. Furthermore, this can be useful not only for medical students but also for the education of young physicians and endoscopists.

## Conclusions

Practical endoscopic education using a GoPro not only helps prevent infection, but it also helps students learn from the doctor’s point of view. This is considered an effective educational tool that not only increases students’ understanding of endoscopic practice but also stimulates interest among students and helps visualize themselves as future as doctors.

## Data Availability

The datasets used and/or analysed during the current study are available from the corresponding author on reasonable request.

## Abbreviations

COVID-19: Coronavirus disease 2019
EGD: Esophagogastroduodenoscopy

## References

[1] Khabaz Mafinejad M, Mirzazadeh A, Peiman S, Khajavirad N, Mirabdolhagh Hazaveh M, Edalatifard M, Allameh SF, Naderi N, Foroumandi M, Afshari A (2016). Medical students’ attitudes towards early clinical exposure in Iran. Int J Med Educ.

[2] Crumlish CM, Yialamas MA, McMahon GT (2009). Quantification of bedside teaching by an academic hospitalist group. J Hosp Med.

[3] Sorrentino S (2009). Guidelines for bedside teaching. Am J Med.

[4] Zhu N, Zhang D, Wang W, Li X, Yang B, Song J, Zhao X, Huang B, Shi W, Lu R (2020). A novel coronavirus from patients with Pneumonia in China, 2019. N Engl J Med.

[5] Ferrel MN, Ryan JJ (2020). The impact of COVID-19 on Medical Education. Cureus.

[6] Ahmed H, Allaf M, Elghazaly H (2020). COVID-19 and medical education. Lancet Infect Dis.

[7] Wong TW, Lee CK, Tam W, Lau JT, Yu TS, Lui SF, Chan PK, Li Y, Bresee JS, Sung JJ (2004). Cluster of SARS among medical students exposed to single patient, Hong Kong. Emerg Infect Dis.

[8] Sandhu P, de Wolf M (2020). The impact of COVID-19 on the undergraduate medical curriculum. Med Educ Online.

[9] Gu J, Han B, Wang J. COVID-19: gastrointestinal manifestations and potential fecal-oral transmission. Gastroenterol 2020, 158(6):1518–9. 10.1053/j.gastro.2020.02.054 PMC7130192 32142785

[10] Xiao F, Tang M, Zheng X, Liu Y, Li X, Shan H. Evidence for gastrointestinal infection of SARS-CoV-2. Gastroenterol 2020, 158(6):1831–3 e1833. 10.1053/j.gastro.2020.02.055 PMC7130181 32142773

[11] Siau K, Hodson J, Neville P, Turner J, Beale A, Green S, Murugananthan A, Dunckley P, Hawkes ND (2020). Impact of a simulation-based induction programme in gastroscopy on trainee outcomes and learning curves. World J Gastrointest Endosc.

[12] Mahmood SU, Crimbly F, Khan S, Choudry E, Mehwish S (2020). Strategies for rational use of personal protective equipment (PPE) among Healthcare Providers during the COVID-19 Crisis. Cureus.

[13] Koh W, Khoo D, Pan LTT, Lean LL, Loh MH, Chua TYV, Ti LK (2020). Use of GoPro point-of-view camera in intubation simulation-A randomized controlled trial. PLoS ONE.

[14] Navia A, Parada L, Urbina G, Vidal C, Morovic CG (2021). Optimizing intraoral surgery video recording for residents’ training during the COVID-19 pandemic: comparison of 3 point of views using a GoPro. J Plast Reconstr Aesthet Surg.

[15] Araujo FJO, de Lima LSA, Cidade PIM, Nobre CB, Neto MLR (2020). Impact of Sars-Cov-2 and its Reverberation in Global Higher Education and Mental Health. Psychiatry Res.

[16] Sahu P (2020). Closure of Universities due to Coronavirus Disease 2019 (COVID-19): impact on Education and Mental Health of students and academic staff. Cureus.

[17] Wang J, Du G. COVID-19 may transmit through aerosol. Ir J Med Sci 2020, 189(4):1143–4. 10.1007/s11845-020-02218-2 PMC7094991 32212099

[18] Dolinger MT, Kumta NA, Greenwald DA, Dubinsky MC (2020). Outcomes of Universal Preprocedure Coronavirus Disease 2019 Testing before Endoscopy in a Tertiary Care Center in New York City. Gastroenterology.

[19] Ueyama H, Akazawa Y, Fujisawa T, Isayama H, Nagahara A (2022). A novel endoscopic shield: a barrier device to minimize virus transmission during endoscopy. Endoscopy.

[20] Chiu PWY, Ng SC, Inoue H, Reddy DN, Ling Hu E, Cho JY, Ho LK, Hewett DG, Chiu HM, Rerknimitr R (2020). Practice of endoscopy during COVID-19 pandemic: position statements of the Asian Pacific Society for Digestive Endoscopy (APSDE-COVID statements). Gut.

[21] Bang G, Kwon OY (2022). Real-time online point-of-view filming education for teaching clinical skills to medical students. Korean J Med Educ.

